# Genome-Wide Identification and Expression Analysis of SNARE Genes in *Brassica napus*

**DOI:** 10.3390/plants11050711

**Published:** 2022-03-07

**Authors:** Jing Xu, Xu Zhao, Jiandong Bao, Yanan Shan, Mengjiao Zhang, Yanan Shen, Yakubu Saddeeq Abubakar, Guodong Lu, Zonghua Wang, Airong Wang

**Affiliations:** 1 State Key Laboratory of Ecological Pest Control for Fujian and Taiwan Crops, Fujian Agriculture and Forestry University, Fuzhou 350002, China; jingxu0217@163.com (J.X.); dature_508@163.com (X.Z.); shanyanan2022@163.com (Y.S.); z1932737435@163.com (M.Z.); shenyanan0925@163.com (Y.S.); lgd@fafu.edu.cn (G.L.); 2 Haixia Institute of Science and Technology, Fujian Agriculture and Forestry University, Fuzhou 350002, China; 3 College of Life Science, Fujian Agriculture and Forestry University, Fuzhou 350002, China; baojd@fafu.edu.cn (J.B.); ay.saddeeq@yahoo.com (Y.S.A.); 4 Institute of Oceanography, Minjiang University, Fuzhou 350108, China

**Keywords:** *Brassica napus*, SNARE, gene family, *Sclerotinia sclerotiorum*, expression profile, hormone stimuli

## Abstract

SNAREs (soluble *N*-ethylmaleimide-sensitive factor attachment protein receptors) are central components that drive membrane fusion events during exocytosis and endocytosis and play important roles in different biological processes of plants. In this study, we identified 237 genes encoding SNARE family proteins in *B. napus* in silico at the whole-genome level. Phylogenetic analysis showed that BnaSNAREs could be classified into five groups (Q (a-, b-, c-, bc-) and R) like other plant SNAREs and clustered into twenty-five subclades. The gene structure and protein domain of each subclade were found to be highly conserved. In many subclades, *BnaSNAREs* are significantly expanded compared with the orthologous genes in *Arabidopsis thaliana*. *BnaSNARE* genes are expressed differentially in the leaves and roots of *B. napus*. RNA-seq data and RT-qPCR proved that some of the *BnaSNAREs* are involved in the plant response to *S. sclerotiorum* infection as well as treatments with toxin oxalic acid (OA) (a virulence factor often secreted by *S. sclerotiorum*) or abscisic acid (ABA), methyl jasmonate (MeJA), and salicylic acid (SA), which individually promote resistance to *S. sclerotiorum*. Moreover, the interacted proteins of BnaSNAREs contain some defense response-related proteins, which increases the evidence that BnaSNAREs are involved in plant immunity. We also found the co-expression of BnaSYP121/2s, BnaSNAPs, and BnaVAMP722/3s in *B. napus* due to *S. sclerotiorum* infection as well as the probable interaction among them.

## 1. Introduction

Vesicle trafficking is essential for diverse biological processes including cell polarity, growth, development, and adaptation [1,2,3,4,5]. The cargo exchange of trafficking vesicles promotes the vesicle-mediated communication among eukaryotic cells through the exocytic and endocytic pathways. These exocytic and endocytic processes are dependent on the targeted membrane fusion of vesicles that delivers membranes, proteins, and soluble cargos between subcellular membranous compartments and the plasma membrane [6]. This membrane fusion mechanism is highly conserved in all eukaryotes [7] and the central components driving the actual membrane fusion events are a set of proteins dubbed as SNAREs (soluble *N*-ethylmaleimide-sensitive factor attachment protein receptors) [8].

SNAREs can be classified into v-SNAREs (associated with transport vesicles) and t-SNAREs (associated with the target compartments) [9]. Considering that some SNARE proteins may have multiple functions, another classification method is generally accepted: Qa-, Qb-, Qc-, Qbc-, and R-SNAREs according to the central amino acid present in the hydrophobic heptad repeats of the proteins [10]. The fusion of vesicles with their target membrane is driven by a cluster of four coiled-coil helices, termed Qa, Qb, Qc, and R, each of which is contributed by three or four individual SNARE proteins (a single Qbc-SNARE protein carries two SNARE helices: Qb and Qc) [11]. The specific interaction between v-(R)SNARE and a cognate set of t-(Q)SNAREs is an important part of the mechanisms that partly influence the accuracy of the transport. SNARE proteins form a superfamily of diverse proteins with at least 64 members in *Arabidopsis thaliana* [11], 60 members in rice [11], 63 members in tomato [12], 69 members in *Populus trichocarpa* [13], and 173 members in wheat [14]. Compared to other eukaryotes, plants so far have the highest number of identified SNAREs; *Homo sapiens* has 38 SNAREs [15], *Drosophila melanogaster* has 26 [16], and there are between 21 and 25 of them in *Saccharomyces cerevisiae* [17,18]. Sansebastiano and Piro were of the opinion that this increase in the number of SNAREs in plants was a result of gene expansion of partially redundant genes in conserved subfamilies and not the evolution of new isoforms [13]. The expansion of SNARE encoding genes in plants implies the importance of this superfamily during the growth and development of the plants, as well as for biotic and abiotic stress responses [19].

During the last 20 years, evidence for the diverse functions of SNAREs at multiple stages of plant development rapidly accumulated. At the cellular level, SNARE proteins express in diverse organelles such as the plasma membrane, ER, transport vesicles, Golgi apparatus, and trans-Golgi network (TGN). The proteins are previously reported to mediate the processes of vacuole biogenesis, vacuolar transport, vesicle fusion, secretion, cell growth, and ion homeostasis. AtVAMP721 interacts with the potassium channels AtKAT1 and AtKC1 to maintain the currency of the K^+^ channels in *A. thaliana* [20]. Members of AtSYP4 (AtSYP41, AtSYP42, AtSYP43) localize on the same TGN compartment and maintain the morphology of both the Golgi apparatus and TGN [21]. The homodimer form of the ER-localized R-SNARE protein AtSEC22 plays a major role during anterograde and retrograde transports by promoting efficient membrane fusion and assisting in the assembly of higher-order complexes. Furthermore, the Qc-SNARE AtBET12 together with the Qb-SNARE AtMEMB12 negatively regulates the secretion of pathogenesis-related protein 1(PR1) in *A. thaliana* [22].

At the tissue level, several types of SNAREs were reported to play vital roles in root growth, pollen tube growth, and seed maturation. For example, the membrane-localized Qa-SNARE *AtKNOLLE* (*AtSYP111*) is highly expressed in organs containing dividing cells and is specifically involved in cytokinetic vesicle fusion [23]. *AtSYP123* is expressed and accumulated in the cells present at the tip region of root hairs during root development, while *AtSYP124*, *AtSYP125*, and *AtSYP131* only express in pollen and are involved in pollen tube growth [24].

At the whole-plant level, SNAREs are mainly activated in response to stresses such as drought/osmotic stress, high salinity, abscisic acid (ABA)-induced stress, and pathogen stimuli. For example, AtSYP121 is involved in ABA-dependent drought stress in tobacco and non-host resistance against powdery mildew as well as oomycete attack in *A. thaliana* [2,23,25]. As a paralog of AtSYP121, AtSYP122 is phosphorylated in response to the elicitor flagellin [26] and has redundant functions with AtSYP121 in plant immunity and general secretion events [27,28]. Another Qa-SNARE, SYP132 also plays roles in bacterial defense and symbiosome definition in *Nicotiana benthamiana* and *Medicago truncatula*, respectively [29,30]. AtSYP61 plays an important role in osmotic stress tolerance and the ABA-dependent regulation of stomatal responses [31]. ShNPSNl11 plays a positive role in defense activation and host resistance to *Oidium neolycopersici* in tomato [25].

*Brassica napus* is a major oil crop in temperate regions of the world. It belongs to the family *Brassicaceae*. The amphidiploid *B. napus* (2n = 38, AACC) was formed as a hybrid between progenitors of *B. rapa* (2n = 20, AA) and *B. oleracea* (2n = 18, CC) ~7500 years ago [32], both of which underwent whole-genome triplication [33,34]. More ancient polyploidization events [35,36] along with the recent hybridization and subsequent gene loss shaped the *B. napus* genome and determined the size of the entire gene complement ~100,000 genes as well as the individual gene families [37]. This evolutionary process and the close relationship of *B. napus* with *A. thaliana* make *B. napus* an ideal material for gene family evolutionary research. However, the number, nature, general relationships, and functions of the various SNARE proteins present in *B. napus* remain in the dark. Therefore, in this study, we first used an in silico approach to carry out global identification of the members of the *SNARE* family in *B. napus* and then systematically analyze their structural similarities, evolutionary relationships, and transcriptional profiles under the influences of the necrotrophic fungus *Sclerotinia* *sclerotiorum*, oxalic acid (OA), methyl jasmonate (MeJA), salicylic acid (SA), and abscisic acid (ABA).

## 2. Results

### 2.1. Identification of SNARE Genes in B. napus

To identify all members of the SNARE family in *B. napus*, three methods including Pfam analysis, conserved domains search, and orthologous sequence BLAST were used. A total of 237 *BnaSNAREs* were identified (Table S1 and Figure S1). Of these genes, six (6) couples sharing 100% identity on the amino acid level but different nucleotide sequences were considered to be different *BnaSNAREs*. All candidate *BnaSNAREs* were named according to their best hit in *Arabidopsis*. Each gene name starts with an abbreviation for the species name *B. napus (Bna)*, followed by the name of the most prominent *Arabidopsis* gene from this subclade (e.g., “*BnaSYP122*” for *AtSYP122*-like genes, “*BnaSEC20*” for *AtSEC20*-like genes, “*BnaYKT61*” for *AtYKT61*-like genes). Exceptions are *BnaSNAP33s*, *BnaSNAP30s*, and *BnaSNAP29s* which are named according to their protein molecular weight (e.g., “BnaSNAP31” represents the protein molecular weight is approximate 31 kDa). Genes on different chromosomes belonging to the same subclade were consecutively numbered according to their chromosome number from low-to-high values (e.g., four *KNOLLE*-like genes *BnaC05g06210D*, *BnaA06g04950D*, and *BnaA08g26870D*, *BnaC08g13620D* were named separately as “*BnaKNOLLEa*”, “*BnaKNOLLEb*”, “*BnaKNOLLEc*”, and “*BnaKNOLLEd*”). In the case of *SYP4s*, our phylogenies did not provide clear orthologous relationships among *SYP41s*, *SYP42s*, and *SYP43s* genes from *B. napus* and *Arabidopsis*. We therefore named the *SYP4* subclade genes of *B. napus* as *BnaSYP44*, *BnaSYP45*, and *BnaSYP46*, taking up the current code of *Arabidopsis*. A similar strategy was adopted to name *USE1*, *SFT1*, *MEMB1*, and *YKT62-like* genes. *VAMP724*, *VAMP726*, and *VAMP728* genes were similar to the case of *BnaSYP4s*, but the strategy did not fit this case because the codes in *Arabidopsis* are up to eight (*VAMP728*). “Slash” was rather used in naming these genes (e.g., “*BnaVAMP724/6/8a*”, “*BnaVAMP724/6/8b*”).

Almost all of the identified *SNAREs* in *B. napus* showed the same conserved domain with their respective orthologs in *Arabidopsis* according to NCBI batch CD search (Tables S1 and S2), except for the 11 SYP6-like proteins, which just contain 1 N-terminal syntaxin-6 (PF09177) domain but lost a C-terminal SNARE domain. Furthermore, of these 11 SYP6-like proteins from *B. napus*, 3 orthologs from *Arabidopsis* were not previously identified. Considering the fact that the conserved N-terminal syntaxin-6 domain is unique to the SNARE family, we believe that the 11 genes belong to the SNARE family and we classified them as SYN-sub-family.

The BnaSNARE proteins have varying physicochemical characteristics (Table S1). Isoelectric points (pIs) of the proteins are between 4.44 and 11.84, and their molecular weights (MWs) range from 9.02 to 120.51 kDa. Exceptions are BnaSYP31d with 7.15 kDa, and both BnaSYP112e and BnaSYP112f have MW of 8.05 kDa, which fall below the range; BnaVTI11e, however, has an MW of 184.07 kDa which falls above the stated range. Subcellular localization prediction for the BnaSNARE proteins indicated that they are localized at the plasma membrane, ER, Golgi, vacuole, and a small group were located in the cytoplasm, mitochondrion, and nucleus (Table S3).

### 2.2. BnaSNAREs Belong to Well-Defined Subfamilies That Were Correlated to Their Gene Structures and Conserved Motifs

**Figure 1:**
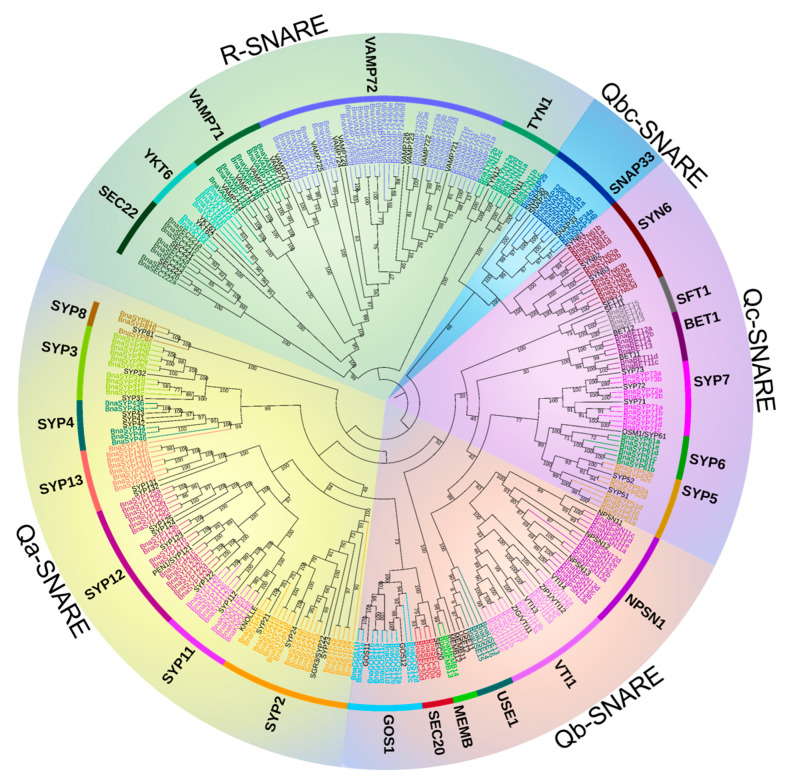
Maximum-likelihood phylogeny of SNARE proteins from *B. napus* and *A. thaliana*. A phylogenetic tree of SNARE proteins from *B. napus* and *A*. *thaliana* was constructed using IQ-TREE. The colored *B. napus* genes are subclade-specific, whereas *A. thaliana* genes were in black. Subfamilies were indicated using *A. thaliana* gene names, and sub-family names according to priority rule [11] were shown in brackets if different from the *A. thaliana* gene names. Despite the absence in other research, the *A. thaliana* genes *SYN61*, *SYN62*, and *SYN63* and their orthologous in *B.* *napus* were included in the phylogeny. A version of the tree with untransformed branches and including the accession numbers can be found in Figure S2.

A maximum-likelihood phylogenetic tree of all the *SNARE* genes from *A. thaliana* and *B. napus* shows that the *B. napus* genome retains all the orthologs of *A. thaliana SNAREs* and the gene phylogeny roughly followed species phylogeny. In several subclades, one *SNARE* in *A. thaliana* is closely related to a double of two *B. napus* homologs (e.g., *SFT1*, *USE1* Figure 1 and Table S4) which is consistent with the chromosome multiples of *B. napus* and *A. thaliana* as *B. napus* is heterotetraploid while *A. thaliana* is diploid. In many subclades, *SNARE* homologs in *B. napus* are significantly expanded compared to those in *A. thaliana*. Measured from the total point of view, the number of *SNAREs* in *B. napus* is much more than double of those in *A. thaliana*, in fact, nearly four times (e.g., *TYN1*, *SEC20*, *SYP5* subclades Figure 1 and Table S1). The topology in *BnaSYP12*, *BnaYKT6*, *BnaSYP6*, and *BnaVAMP72* subclades (Figure 1 and Table S1) is more complex, suggesting multiple duplication events, before and/or after polyploidization of *B. napus*. The BnaSNARE proteins displayed the same five groups described previously (Q (a-, b-, c-, bc-) and R) in *A. thaliana* and presented a similar proportion of members compared to *A. thaliana* (Table S5). Therefore, Qa-, Qb-, and Qc-BnaSNAREs are composed of 69, 44, and 37 (+11) genes, respectively. Qbc-BnaSNAREs have 10 members and R-BnaSNAREs have 65 members.

**Figure 2:**
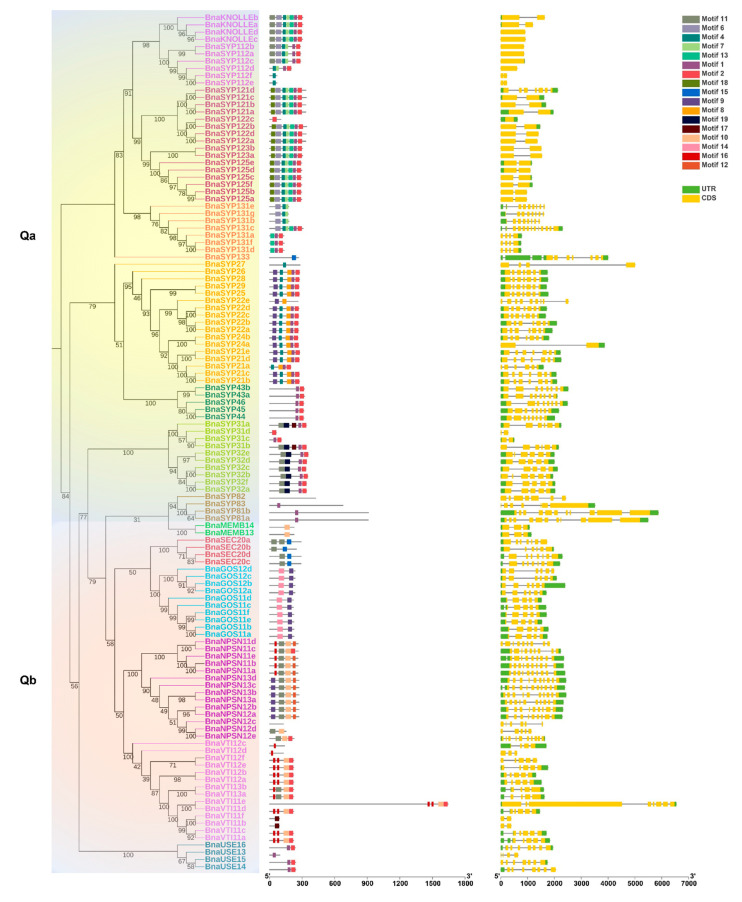

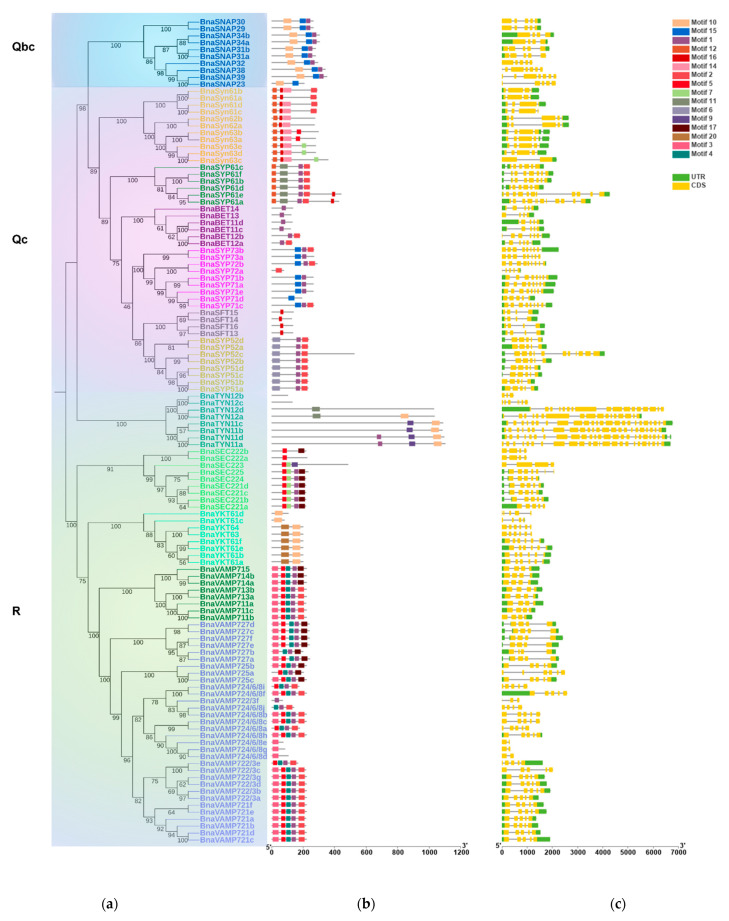
Gene intron/exon structures and protein conserved motifs of BnaSNAREs. (**a**) Phylogenetic tree of BnaSNARE proteins. (**b**) Conserved motif arrangements of BnaSNAREs. Twenty conserved motifs labeled with different colors were found in the BnaSNAREs sequences using the MEME program. Sequences of the conserved motif are presented in Table S5. (**c**) Exon-intron organizations of *BnaSNAREs*. The green boxes represent 5′or 3′ untranslated regions, yellow boxes represent exons, and black lines represent the introns. The lengths of the exons and introns can be determined by the scale at the bottom.

The conserved motifs of each BnaSNARE protein sequence were identified by MEME and analyzed with the InterProScan tool (Figure 2b and Table S6). In brief, proteins in the same subclade seemed to share a similar motif composition, corresponding to the phylogenetic classification of BnaSNARE proteins. Motifs one and two correspond to the SNARE domains found in both Q- and R- SNARE proteins. Motifs six, seven, and eight, were found to be related to the syntaxin domains present in Q-SNAREs of *B.* *napus*, while motifs three, four, five, sixteen, and eighteen were found to be related to the Synaptobrevin and Longin domains present in R-SNAREs. In addition, motif fifteen is specific to Qbc SNARE, while motif nineteen is just present in *BnaSYP3s*. Along with the conserved motifs, the distribution of introns and exons in the 237 *BnaSNAREs* was analyzed with GSDS 2.0 (Figure 2c). We found a conserved number of introns within the subclades which is consistent with the phylogenetic classification. In detail, *Qa-SNAREs* contain various introns between 0 and 11. Among them, *BnaSYP11s* and *BnaSYP12s* contain the minimal introns 0 or 1. *BnaSYP13s* contain the most variable number of introns which are from 3 to 11. Almost all of the *BnaSYP2s* had six introns with two exceptions: *BnaSYP22e* and *BnaSYP27* having eight and three introns, respectively. A similar situation also occurred in other types of *BnaSNAREs* (Figure 2c). *Qb-SNAREs* contain various introns between 1 and 9; *Qc-SNAREs* contain 3 to 11 introns; *Qbc-SNAREs* contain 3, 4, or 6 introns while *R-SNAREs* contain 1 to 23 introns. More so, *R-SNAREs*, *BnaTYN11a*, *BnaTYN11b*, *BnaTYN11c*, and *BnaTYN11d* harbor 23 introns each, which is the largest number detected in all the *SNAREs* in *B. napus*.

### 2.3. BnaSNAREs Exhibit a High Rate of Homolog Retention and Gene Duplication in the Genome

**Figure 3:**
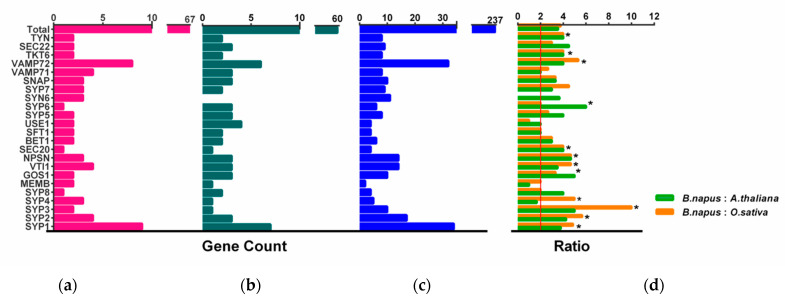
Number of *SNARE* genes. (**a**–**c**). The number of *SNARE* genes identified per *SNARE-type* subgroup in (**a**) *A. thaliana*; (**b**) *O. sativa*, and (**c**) *B. napus* [11]. (**d**) The ratio of total *SNARE* gene numbers to those in all subgroups is shown for *B. napus*: *O. sativa* (green) and *B. napus*: *A. thaliana* (orange). The expected ratio (2:1) in (**d**) is indicated by a red vertical line, and asterisks mark a significant deviation from the expected value (χ^2^ test, * *p* < 0.05).

In many land plants, the number of identified *SNAREs* is between 50 and 70 [11,14]. Rice and *Arabidopsis*, which are both diploid, have a similar number of *SNARE genes* (60 and 64, respectively), while 173 *SNAREs* were found in wheat. The number of *SNAREs* in wheat is expected to be high considering the fact that wheat is an allohexaploid plant (60*3 = 180) (Table S5). *B. napus* contained the highest number of *SNAREs* in characterized land plant species and algae. Fifteen out of a total of twenty-five subclades contain significantly more than two times the number in rice and *Arabidopsis* (Figure 3, *t*-test, *p* < 0.001) and approach four times on the whole level (*t*-test, *p* = 0.11, 0.62).

To better understand why *SNARE*s are so abundant in the *B. napus* genome, we analyzed in detail the homologous pairs from the A and C sub-genomes (Table 1). More than three-quarters (76.8%) of the 237 *BnaSNARE* genes identified are present in double (even as high as 89.7% when the “not categorized” gene is removed) (Table 1 and Table S4).

**Figure 4:**
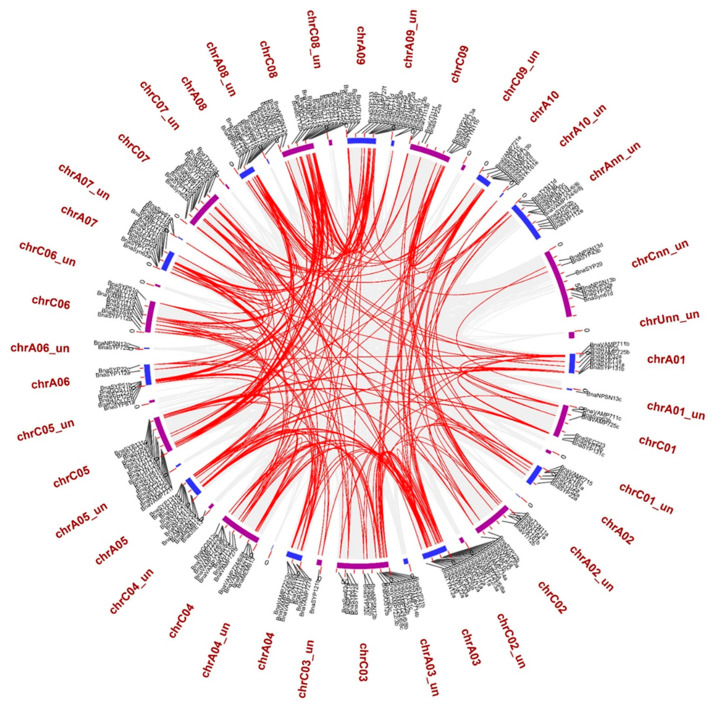
The locations on chromosomes and homologous relationships of the *BnaSNAREs*. All *BnaSNAREs* were mapped to their respective locus in the *B.* *napus* genome in a circular diagram using Advanced Circos of TBtools. An and Cn sub-genomes are indicated by shades of blue and purple colors.

We mapped the 237 *BnaSNARE*s on 10 An and 9 Cn chromosomes and found that they are unevenly distributed in *B. napus*. A total of 121 *BnaSNARE* genes were mapped on the An sub-genome, while 116 were mapped on the Cn sub-genome (Figure 4 and Table S1). Chromosomes A03 (17) and C03 (19) contain the highest number of *BnaSNARE*s in the An and Cn sub-genomes, respectively. The lowest number of *BnaSNAREs* was found on chromosomes A02 and C01 where each contains six. The remaining chromosomes harbor between 7 and 18 *BnaSNAREs*. Additionally, 11 and 8 members of the *BnaSNAREs* from A and C sub-genomes, respectively, could not be mapped to a particular chromosome. Based on their chromosome positions, we identified 22 clusters of tandem duplication cases, including 47 *BnaSNARE* genes (47/237, 19.8%) (Table S7), suggesting a role of tandem duplication events in the expansion of *BnaSNARE* gene members.

The duplication analysis showed explosive gene duplication events (288 duplication events including 206 *BnaSNARE* genes) in *BnaSNAREs* (Table S8). The sub-family *BnaVAMP72*s have the largest number of duplication events (55/288, 19.1%) in the *B. napus* genome, followed by *BnaSYP1*s (35/288, 12.2%) (Figure 4 and Table S8). To determine the possible selection constraints on the duplicated *BnaSNARE*s, we estimated the Ka/Ks ratio for each pair of paralog genes (Table S8). We found that the Ka/Ks ratios of the duplicated *BnaSNAREs* gene pairs are all less than 1. Besides, the Ka/Ks value of 97.9% (282/288) duplicated gene pairs is less than 0.5, suggesting a high level of purifying selection stress of evolution.

### 2.4. Differential Expression of BnaSNAREs in Leaves and Roots of B. napus

To understand the biological functions of *BnaSNAREs*, we first detected the expression profiles of *BnaSNARE* genes in leaves and roots of *B. napus* (Table S9 and Figure S2). The RPKM values of *BnaSNAREs* were downloaded from genoscope (http://www.genoscope.cns.fr/brassicanapus/, accessed on 26 October 2020). *BnaSNAREs* were expressed differentially in the roots and leaves of *B. napus.* Most of the root-expressed genes were expressed unequally in leaves (e.g., *BnaSNAPs* and *BnaSYP12s* were highly expressed in roots but not in leaves). In total, expression levels of more than half of the BnaSNARE genes were higher in root than in leaves. The RPKM of 37 *BnaSNAREs* was drastically low and negligible expression was recorded in roots, leaves, or neither. The *BnaSYP1*s contributed the most low-expressed genes followed by the *BnaVAMP7*s. The reason for this low expression was probably due to tissue specificity in the expression or the presence of processed pseudogenes.

### 2.5. BnaSNAREs Are Involved in Regulation of B. napus Resistance to S. sclerotiorum

**Figure 5:**
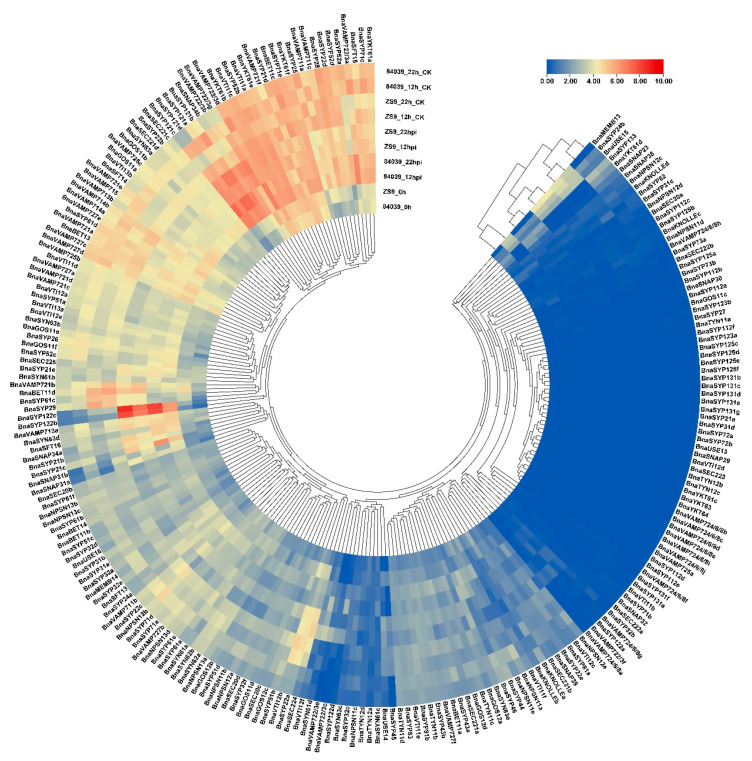
Expression of *BnaSNAREs* in response to the infection of *S. sclerotiorum*. The transcription level of *BnaSNAREs* in leaves at 12 h and 22 h post-inoculation with *S. sclerotiorum* is shown as a heatmap. Data were normalized by log2 (FPKM+1). The cluster tree of the *BnaSNARE* genes based on the expression level is shown at the center and top.

*S. sclerotiorum* is one of the main pathogens causing serious stem rot disease of *B. napus* [38]. To improve our understanding of the role of *BnaSNAREs* during the infection of *S. sclerotiorum*, we inoculated a resistant variety Zhongshuang9 and a susceptible variety 84039 and collected samples at 0 hpi, 12 hpi, and 22 hpi, then performed RNA extraction, cDNA library construction, and sequencing. The RNA-seq data showed that the biotic stress caused by *S.* *sclerotiorum* resulted in the accumulation of *BnaSNARE* transcripts (Figure 5 and Table S10). The expression levels of the well-known resistance-related Qa-SNARE *SYP121(pen1)* orthologs, *BnaSYP121s* and *BnaSYP122s*, were highly up-regulated both in varieties 84039 and Zhongshuang9 at 12 h and even higher at 22 h after inoculation with *S.* *sclerotiorum*. Furthermore, other genes belonging to *BnaSYP21s*, *BnaSYP32s*, *BnaSYP52s*, *BnaSNAP33s*, *BnaGOS11s*, *BnaVTI12s*, *BnaVAMP7s*, and *BnaSYN6s* were also up-regulated after inoculation with *S. sclerotiorum*. Among them, *BnaSYP21b*, *BnaSYP21c*, *BnaSYP32b*, *BnaSYP61d*, *BnaSYP63d*, *BnaSNAP31a*, *BnaSNAP31b, BnaSNAP34a*, *BnaSNAP34b,* and *BnaVAMP722/3s* were up-regulated in both 84039 and Zhongshuang9 varieties, while *BnaSYP22b*, *BnaSYP32d*, *BnaMEMB13*, *BnaGOS11b*, and *BnaUSE14* were mainly up-regulated in the resistant variety Zhongshuang9 and some other genes as *BnaSYP43a* and *BnaSYN61c* were up-regulated in the susceptible variety 84039. Some *BnaSNARE* genes were down-regulated in response to inoculation with *S. sclerotiorum,* such as *BnaKONLLEa*, *BnaSYP83, BnaVTI11f*, *BnaNPSN12b*, *BnaNPSN12e*, and *BnaSYN62s*. Almost all members of *BnaSYN61s* and *BnaSYN63s* genes showed a trend becoming up-regulated in both 84039 and Zhongshuang9 varieties in response to *S. sclerotiorum*, while *BnaSYN62s* were down-regulated, implying that *BnaSYN6s* are associated with plant resistance.

**Figure 6:**
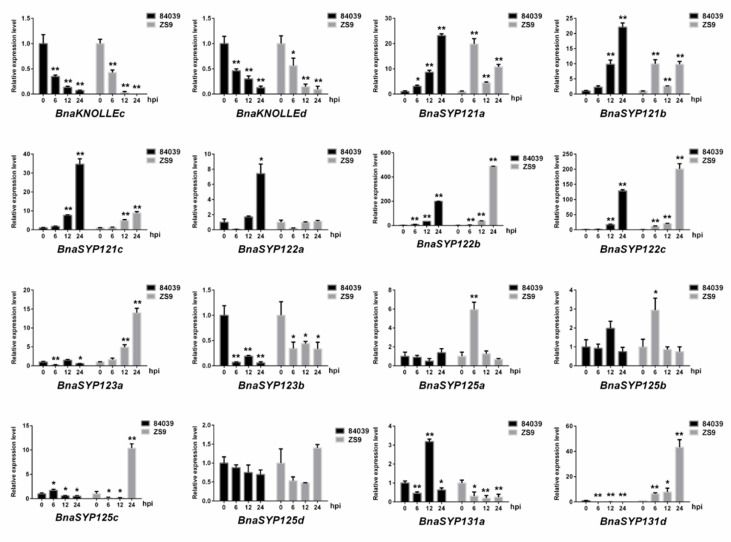
Expression of *BnaSYP1-type* genes in resistant variety Zhongshuang9 and susceptible variety 84039 of *B. napus* at different times after inoculation with *S. sclerotiorum*. RT-qPCR data were calculated by the methods of 2^−ΔΔCT^. STDEV is indicated as error bars. All data are the mean of 3 biological replicates. Significant differences are shown by * (*p* < 0.05) and ** (*p* < 0.01).

To further verify the above results, we performed real-time quantitative reverse transcription-PCR (RT-qPCR) to detect the expression level of *BnaSYP1s* (Figure 6). Sixteen of the tested *BnaSYP1s* showed either induction or suppression in response to *S. sclerotiorum* infection, while others had too weak transcript abundances to detect. The expression levels of *BnaKNOLLEc* and *BnaKNOLLEd* were decreased at all time points and reached the lowest level at 24 h. On the contrary, *BnaSYP121s* and *BnaSYP122s* were highly up-regulated. The expression of *BnaSYP125s* changed slightly at each time point in variety 84039 but these changes were highly pronounced in the Zhongshuang9 variety. Several genes belonging to one subclade showed opposite expression level trends: *BnaSYP123a* was up-regulated while *BnaSYP123b* was down-regulated in both varieties; *BnaSYP131a* was up-regulated in variety 84039 but down-regulated in variety Zhongshuang9 while the reverse was the case for *BnaSYP131d* gene expression.

### 2.6. BnaSYP1s Respond to Phytohormones and Oxalic Acid Treatments

**Figure 7:**
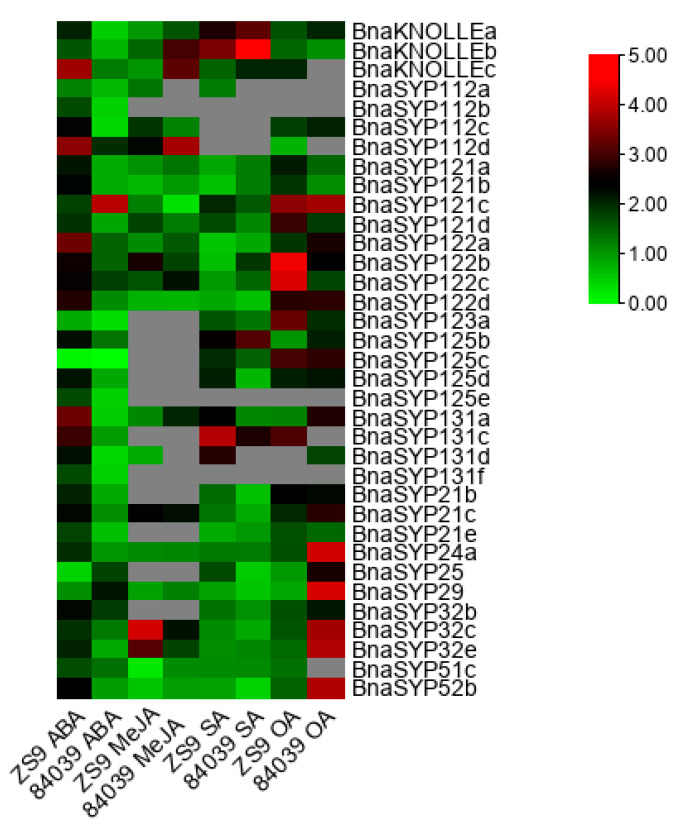
Expression profiles of Qa-*BnaSNAREs* in resistant variety Zhongshuang9 and susceptible variety 84039 of *B. napus* under treatment with ABA, MeJA, SA, and OA. Green and red colors are used to represent low-to-high expression levels, and colors scales correspond to the fold-change values compared with the counterpart control. RT-qPCR data were calculated by the method of 2^−ΔΔCT^ and appeared after Log2 conversion. The gray color represents no available data. All values were detailed in Table S11.

*SNAREs* were found to participate in plant development [1,3,29], abiotic stress [12,31], and pathogen resistance pathways [21,25,30,39,40,41]. Furthermore, MeJA, SA, and ABA were proved to regulate plant resistance to *S. sclerotiorum* [42,43,44,45,46]*. B. napus* varieties Zhongshuang9 and 84039 were treated with exogenous OA, MeJA, SA, and ABA. RT-qPCR results showed that most of the tested genes from BnaSYP1, BnaSYP2, BnaSYP3, and BnaSYP52 were sensitive to all treatments (Figure 7 and Table S11). Most of the tested genes showed notably increased expression levels to a large degree in *B. napus* variety Zhongshuang9 after treatment with ABA, while several genes were up-regulated to more than 2-fold in variety 84039. After treatment with MeJA, transcripts from many genes could not be detected. A few genes such as *BnaSYP112c*, *BnaSYP121d*, *BnaSYP122b*, *BnaSYP21c*, *BnaSYP32c*, and *BnaSYP32e* were induced to a larger degree in the resistant variety than in the susceptible variety; while *BnaKNOLLEs* was significantly down-regulated in the resistant variety compared to the susceptible variety. *BnaSYP121c*, however, was significantly down-regulated in the susceptible variety in comparison to the resistant variety. The SA-induced transcript changes of *BnaKNOLLEs*, *BnaSYP123a*, *BnaSYP125b*, *BnaSYP125c*, *BnaSYP131a*, and *BnaSYP131c* were increased in both resistant and susceptible varieties, while *BnaSYP122a*, *BnaSYP122d*, *BnaSYP21e*, *BnaSYP29*, *BnaSYP32,c* and *BnaSYP52b* were suppressed by SA in both varieties. The expression of *BnaSYP122b* was induced in the susceptible variety but inhibited in the resistant variety whereas *BnaSYP25* showed the opposite trend. *BnaKNOLLEs*, *BnaSYP121a*, *BnaSYP121b*, *BnaSYP121d*, *BnaSYP122d*, *BnaSYP24a*, and *BnaSYP29* showed similar expression pattern as those induced by MeJA.

OA treatments also induced the different accumulation of *BnaSNAREs* transcripts. Most of the tested *BnaSNAREs* were up-regulated by more than two-fold under treatment with OA, especially in the susceptible variety 84039. The expression levels of *BnaSYP24a*, *BnaSYP25*, *BnaSYP29*, *BnaSYP32c*, *BnaSYP32e,* and *BnaSYP52b* increased to a very high level in variety 84039, whereas those of other genes such as *BnaSYP121d*, *BnaSYP122b*, *BnaSYP122c,* and *BnaSYP123a* were highly induced in the resistant variety Zhongshuang9. Besides, transcripts of *BnaKNOLLEa*, *BnaSYP112c*, *BnaSYP121c*, *BnaSYP122d*, *BnaSYP125c*, *BnaSYP125d*, *BnaSYP21b*, *BnaSYP21e*, and *BnaSYP32b* showed similar trends in both 84039 and zhongshuang9 varieties under OA treatment. These results suggest that BnaSNAREs participate in diverse signaling pathways through complicated functional mechanisms.

### 2.7. BnaSYP121/2s Expressions Highly Correlate with Those of BnaSNAPs and BnaVAMP722/3s during S. sclerotiorum Infection

**Figure 8:**
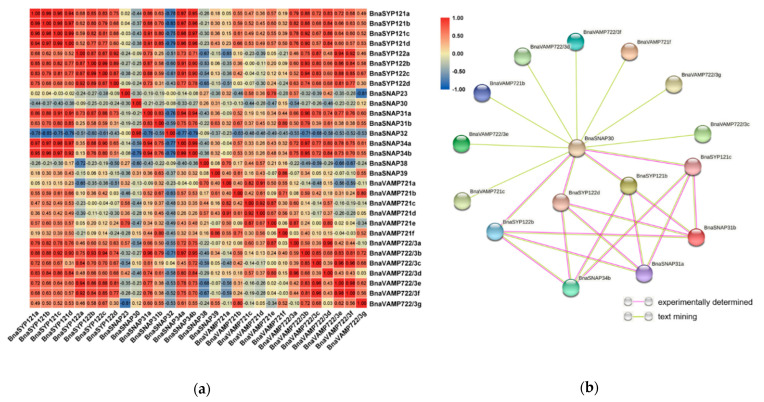
Correlations between the gene expression patterns of BnsSYP121/2s, BnaSNAPs, and BnaVAMP721/2/3s (**a**) and putative interaction networks among these proteins (**b**).

The above results showed that *BnaSYP121/2s* was induced by ABA, MeJA, SA, and OA treatments as well as *S. sclerotiorum* infection, indicating a significant role of BnaSYP121/2s in pathogen resistance. SNARE proteins usually form complexes and play important biological functions in the form of complexes [47]. In addition, it has been reported that SYP121 interacts with the SNARE proteins SNAP33, VAMP721, and VAMP722 to participate in the process of powdery mildew resistance. The correlations of expression patterns among *BnaSYP121s*, *BnaSYP122s*, *BnaSNAPs*, *BnaVAMP721s,* and *BnaVAMP722/3s* during *S. sclerotiorum* infection were analyzed in this paper (Figure 8a). The expression patterns of several genes such as *BnaSNAP31a, BnaSNAP31b, BnaSNAP34a, BnaSNAP34b,* and *BnaVAMP722/3s* showed positive correlations (r ≥ 0.6) with *BnaSYP121/2s*, while *BnaSNAP32* and *BnaSNAP38* showed negative correlations with *BnaSYP122s* (r ≤ −0.6). Several pairs of members among *BnaSYP121s* and *BnaSYP122s* showed a high level of correlation (r ≥ 0.87), which implies the possibility of functional redundancy.

To further uncover the potential association of BnaSYP121/2s, BnaSNAPs, and BnaVAMP722/3s, protein–protein interaction analysis was conducted using STRING-DB (Table S12). Except for 15 undetected proteins, the association among the other 16 proteins is shown in Figure 8b. According to STRING-DB, the interaction patterns of BnaSYP121s and BnaSYP122s with other proteins were exactly the same, and they all interacted with BnaSNAP30, BnaSNAP31a, BnaSNAP31b, and BnaSNAP34b. While BnaVAMP721s and BnaVAMP722/3s only interact with SNAP30, we speculate that SNAP30 may be the key factor in the formation of the SNARE complex. These results showed the co-expression and potential interaction of BnaSYP121/2s, BnaSNAPs, and BnaVAMP722/3s under fungal pathogen infection, suggesting that BnaSYP121/2s may perform anti-fungal functions through the formation of BnaSYP121/2s-SNAPs-VAMP722/3s complexes.

### 2.8. BnaSNAREs Interaction Networks Indicate They Mainly Function in Vesicle-Mediated Transport, Protein Localization, and Response to Abiotic or Biotic Stress

To further investigate the interacted proteins of BnaSNAREs, we built interaction networks of AtSNAREs, and the corresponding orthologs in *B. napus* were identified. A total of 1574 Arabidopsis proteins were found to interact with AtSNAREs (Table S13). By syntenic analysis, 5184 syntenic orthologs were identified in *B. napus* (Table S14). The interaction networks of AtSNAREs showed a very complicated correlation with other proteins including not only the SNARE family members but also many other transport-related proteins, protein receptors, kinases, and so on, which indicate BnaSNAREs are involved in several mechanisms by regulating many downstream factors or being regulated by many upstream proteins.

**Figure 9:**
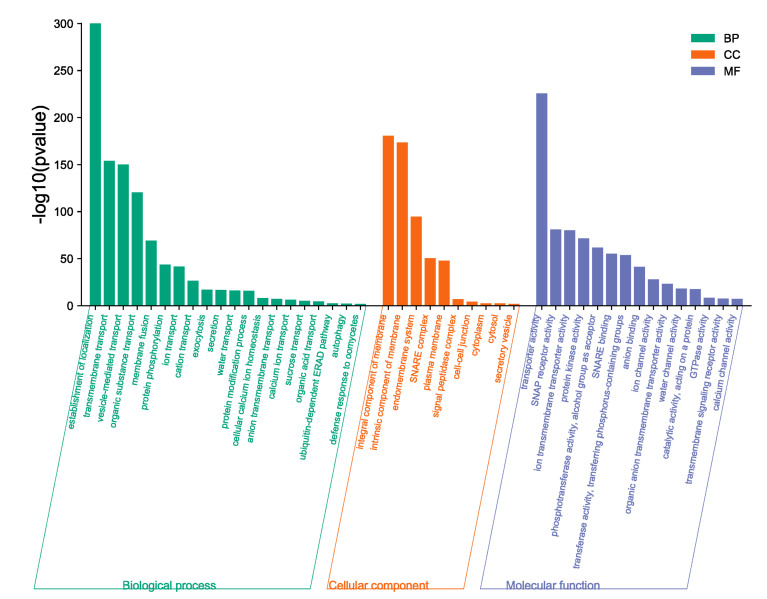
The gene ontology of genes in BnaSNAREs interaction networks.

Gene ontology (GO) enrichment analysis of proteins in BnaSNAREs interaction networks was conducted to reveal their functional characteristics. The most enriched and meaningful BP terms were related to vesicle-mediated transport and localization of proteins (Figure 9), illustrating the conserved functions of SNARE proteins in transporting and localizing proteins in plant cells. Meanwhile, BnaSNAREs were also related to some defense response signaling pathways, such as “defense response to oomycete”, “anion transmembrane transport”, “calcium ion transport”, and “organic acid transport”, indicating that SNARE protein may be involved in plant response to abiotic and biotic stress through association with these proteins. These results will shed light on their undetermined functions in other biological processes.

## 3. Materials and Methods

### 3.1. Identification of SNAREs in B. napus

To identify all candidate members of the SNARE family in *B. napus*, we combined three different methods. First, we identified the conserved domains and Pfams in all of the 64 SNAREs from *A. thaliana* (https://www.arabidopsis.org/, accessed on 23 August 2019) using the online batch CD-search server (https://www.ncbi.nlm.nih.gov/Structure/bwrpsb/bwrpsb.cgi, accessed on 25 August 2019 ). We generated 10 Pfams and 12 conserved domains specific for the SNARE superfamily (Table S2) as previously reported [12]. Then, genome-wide analysis of the Pfams was conducted and proteins containing the above Pfams were considered as SNARE protein candidates. Second, we searched for SNARE conserved domains and Pfams annotation in the *B. napus* genome annotation resources (http://www.genoscope.cns.fr/brassicanapus/, accessed on 26 October 2021) where proteins annotated with any of the 10 Pfams or the 12 conserved domains were identified as SNARE protein candidates. Third, the sequences of the 64 SNARE proteins in *A. thaliana* were used for similarity search against *B. napus* proteome by BLASTp. Finally, all possible BnaSNAREs identified by these three methods were validated by CDD (http://www.ncbi.nlm.nih.gov/Structure/cdd/wrpsb.cgi, accessed on 14 January 2020), Pfam (http://pfam.xfam.org/, accessed on 21 January 2020) and SMART (http://smart.embl-heidelberg.de/, accessed on 16 January 2020) analyses.

Biochemical parameters such as length of sequences, molecular weights, and isoelectric points of BnaSNAREs were calculated by the ProtParam tool (https://web.expasy.org/protparam/, accessed on 19 January 2020) and the subcellular localization was predicted by three different tools: Plant-mPLoc [48], CELLO [49], and pLoc-mEuk [50].

### 3.2. Phylogenetic Analysis

SNARE protein sequences from *B. napus* and *A. thaliana* were aligned using MAFFT [51,52]. Then, a phylogenetic tree was generated using IQ-TREE webserver (http://iqtree.cibiv.univie.ac.at/ accesed on 22 November 2021) [53] as follows: the substitution models were calculated with ModelFinder (integrated with IQ-TREE; best-fit model: JTT+I+G4 chosen according to the Bayesian information criterion) [54]. Subsequently, a phylogenetic tree was generated using Ultrafast bootstraps as well as a Shi-modaira–Hasegawa approximate likelihood ratio test (SH-aLRT) test (1000 replicates each) [55,56,57]. The resulting tree file was visualized with iTOL v 5 [58] (https://itol.embl.de/, accessed on 28 November 2021).

### 3.3. Gene Structure and Protein Conserved Motifs

The gene structure information was displayed in the Gene Structure Display Server [59] (GSDS; http://gsds.cbi.pku.edu.cn, accessed on 5 March 2020). Conserved motifs of these genes were determined using the MEME suite [60] (http://meme-suite.org/tools/meme, accessed on 12 March 2020) with the following parameters: optimum motif widths of 6–50 residues and a maximum of 50 motifs. Then, these motifs were searched using the InterProScan tool [61] (https://www.ebi.ac.uk/interpro/search/sequence-search, accessed on 15 March 2020). The schematic diagram of the amino acid motifs for each SNARE gene was drawn accordingly using Advanced Gene Structure View of TBtools software v1.098 [62].

### 3.4. Chromosomal Spread, Gene Duplication, and Collinear Analysis

The locations on the chromosomes of *BnaSNAREs* were shown by Circos [63]. Detection of putative gene duplication events was done with MCScanX (E-value 10^−5^) [64] and visualized using Advanced Circos of TBtools software v1.098 [62]. Tandem duplication events were defined as two or more homologous genes located on a chromosomal region within 200 kb [65].

### 3.5. Transcriptional Profile of BnaSNAREs in Leaves and Roots

Expression profiles of *BnaSNAREs* in leaves and roots of *B.* *napus* variety “Darmor-bzh” were downloaded from the *B. napus* genome database (https://www.genoscope.cns.fr/brassicanapus/, accessed on 26 May 2020). Reads per kilobase million (RPKM) values of all the *BnaSNAREs* were extracted and submitted to TBtools to generate heatmaps. All of the heatmaps were normalized by Log2 (value).

### 3.6. Plants and Fungal Materials and Growth Conditions

The susceptible (84039) and resistant (Zhongshuang9) varieties of *B. napus* [66] (Figure S3) were provided by Shengyi Liu of the Oil Crops Research Institute, Chinese Academy of Agricultural Sciences. Sclerotia of the fungus *S. sclerotiorum* 1980 were germinated to produce hyphal inoculum on potato dextrose agar (PDA) at 22 °C. The *B. napus* used in these experiments were grown in pots containing soil and vermiculite (3:1 *v*/*v*) under greenhouse conditions at 23–25 °C with a photoperiod of 16/8 h light/dark, fertilization with commercial N:K:P (1:1:1) every 10 d.

### 3.7. Transcriptional Profiling of BnaSNAREs during S. sclerotiorum Infection

After 4 weeks of growth, the 3rd/4th leaves of the *B. napus* were inoculated with agar plugs excised from the edges of growing *S. sclerotiorum* colonies; three biological replicates for the leaf samples were detached at 12 hpi and 22 hpi and sent to Novogene Co., Ltd., (Beijing, China) for RNA extraction, library construction, and transcriptome sequencing using Illumina sequencing platform. After removing the 5′ and 3′-adapters, N > 10% sequences, low-quality sequences (sequence quality values <= Q20), the clean data were aligned to the *B. napus* reference genome (https://www.genoscope.cns.fr/brassicanapus/, accessed on 26 October 2020) using TopHat2 [67] (http://ccb.jhu.edu/software/tophat/index.shtml, accessed on 24 March 2016). The transcript abundance (in RPKM value) of each gene was calculated by HTSeq [68] (https://htseq.readthedocs.io/en/release_0.11.1/, accessed on 24 March 2016).

### 3.8. Plant Treatments, RNA Isolation, and RT-qPCR

The 3rd and 4th leaves of four-week-old Zhongshuang9 and 84039 were sprayed with MeJA, SA, ABA, and OA or inoculated with *S. sclerotiorum* strain 1980 and collected at 0, 6, 12, 24, and 48 h post-inoculation (hpi) and immediately frozen in liquid nitrogen and stored at −80 °C. Three biological replicates for inoculated leaves were maintained.

The total RNA isolation and purification of the samples were performed using RNAprep Pure Plant Plus Kit (Polysaccharides and Polyphenolics-rich) (TIANGEN, Beijing, China). All the RNA isolations for gene expression were done in triplicate for each sample analyzed. RNA integrity was visualized by 1% agarose gel electrophoresis. Their concentrations and purities (OD260/OD280 ratio > 1.95) were determined with a NanoDrop One Microvolume UV-Vis Spectrophotometer (NanoDrop Technologies, Wilmington, DE, USA). Exactly 1 μg of total RNA were reversely transcribed in a 20 μL reaction mixture using PrimeScript™ RT reagent Kit with gDNA Eraser (Takara, Beijing, China) following the manufacturer’s instructions to remove traces of contaminant DNA and prepare cDNA.

Quantitative real-time PCR (RT-qPCR) analysis was used to analyze the expression levels of the identified *BnaSNAREs*. The standard RT-qPCR with SYBR Premix Ex Taq II (TaKaRa, Beijing, China) was repeated at least three times on a CFX96 Real-Time System (BioRad). The results were analyzed using the 2^−(∆∆Ct)^ method, using *BnaACTIN* as the endogenous reference gene [69]. The primers used in this study are listed in Table S15.

### 3.9. Protein–Protein Interaction Analysis and Gene Ontology Analysis

The STRING-DB (v 11.5, https://string-db.org/, accessed on 26 November 2021) online service was used to predict putative protein–protein interaction networks among candidate proteins of BnaSNAREs. The interaction network of AtSNAREs in *A. thaliana* was constructed using the Arabidopsis Interactions Viewer (http://bar.utoronto.ca/interactions/cgi-bin/arabidopsisinteractionsviewer.cgi, accessed on 26 November 2021). Interacted proteins of AtSNAREs were replaced with corresponding syntenic orthologs in *B. napus* using syntenic analysis mentioned above. The GO enrichment analysis of BnaSNAREs interacted proteins was conducted by g: GOSt of g: Profiler (https://biit.cs.ut.ee/gprofiler/gost, accessed on 9 December 2021).

## 4. Discussion

The heterotetraploid nature of *B. napus* and the large size of the BnaSNARE family provide an ideal opportunity to study the evolutionary fates of these genes. The BnaSNARE family (with 237 members) is one of the largest families of proteins among flowering plants [11,12] and has c.3.5 times as many as *Arabidopsis* (237:67~3.5). Another well-analyzed protein family in *B. napus* is NBS-LRR, which has c. 2.9 times as many as *Arabidopsis* (425:149~2.9) [32,70], while fatty acid desaturase family and NAC transcription factor family were expanded to c.3.4 and c.3.6 times as many as in *Arabidopsis*, respectively [71].

In fact, a variety of duplication patterns is observed in some subclades of BnaSNAREs. First, many sequences of *BnaSNAREs* are truncated (e.g., *BnaSYP122b*, *BnaNPSN12c*, *BnaSYP72a*) (Table S1 and Figure 2b,c), indicating that gene amplification occurred through transposable elements. Second, 47 genes from the *BnaSNAREs* are found in close proximity to each other, pointing towards tandem duplications as a mechanism for family expansion (Table S7). Third, each of the two *Arabidopsis SYP2* genes (*AtSYP21* and *AtSYP23*) has five and six paralogous genes in *B. napus* (Table S1). The phylogenetic analysis and gene position on the chromosome of these genes suggests gene duplications in the lineage leading to *Brassica* but before the polyploidization of *B. napus*. It has been found that numerous regions that are homologous to the *Arabidopsis* genome were triplicated within the diploid species of *Brassica* [72,73]. The amphidiploid species *B. napus* has an A genome coming from *B. rapa* and a C genome coming from *B. oleracea.* Furthermore, genes are essentially conserved within these two genomes [73], and this presents a large proportion of segmental or WGD duplication events between An and Cn genomes. We speculate that the gene expansion of SNAREs in *B. napus* is a synergistic effect of polyploidization and hybridization working together.

We found that 90.3% of all the *BnaSNARE* genes were expressed in roots or leaves or both, and the expression was relatively low when compared with the genome-wide assessment [32]. *BnaSYP1s* and *BnaVAMP7s* occupied most of the low transcript abundance genes. Some of these genes may constitute pseudogenes. Because several members of *SYP1s* and *VAMP7s* in other plants were proved to be involved in resistance to biotrophic or semi-biotrophic pathogen infections [2,29,30,39,74], the expressions of *BnaSYP1s* and *BnaVAMP7s* might be induced by certain conditions, thereby making them good candidates for investigating variety-specific resistance to biotic stress. Whether these genes are still functional remains an open question. In some cases, pseudogenes may contribute to the regulation of gene expression and generating genetic diversity by, for example, providing transcription factors (TFs) and RNA polymerase II (Pol II) binding sites [75].

The expression level of genes belonging to the same sub-family is diverse, which may be related to the fact that different members of the same sub-family play diverse roles in the same life activities. In wheat, silencing *TaNPSN11/13* but not *TaNPSN12* reduced resistance to *Puccinia striiformis* f. sp. *tritici* (*Pst*) virulent race CYR2 [41]. In rice, both *OsVAMP711* and *OsVAMP714* belong to the *VAMP71* sub-family; overexpression of *OsVAMP714* enhanced resistance to rice blast while that of *OsVAMP7111* does not [40]. VAMP721/722/724 but not VAMP711/727 are involved in SA-associated apoptosis by interacting with PVA31 to combat pathogen infection [76]. Though having different functions mediated by the same SNARE homologs, functional redundancy has also been reported for these genes. For example, AtSYP123, AtSYP125, and AtSYP131 of the SYP1 sub-family were necessary for the development of male gametophyte in *Arabidopsis* [24]. The VTI1 SNARE family consists of four genes (*AtVTI11*, *AtVTI12*, *AtVTI13*, and *AtVTI14*) in *Arabidopsis* but only *AtVTI11* and *AtVTI12* are expressed at significant levels [77,78,79,80].AtVTI11 and AtVTI12 have different functions in vesicle trafficking to the vacuole, while AtVTI11 and AtVTI12 can functionally substitute each other when forming a complex with AtSYP41 and AtSYP42 to drive vesicle fusion [80,81,82]. Functional overlap was also observed among AtSYP4 family members [82]. AtSYP121/122 form complex with AtSNAP33 and AtVAMP721/722 during defense against powdery mildew, but the complexes so formed with AtVAMP724 and AtVAMP727 are not related to plant immunity [2]. Our RNA-seq data showed similar trends: several members of *BnaVAMP722/3*s were induced by *S. sclerotiorum* infection, while most of the *BnaVAMP727s* members were unaffected.

Diseases caused by *S. sclerotiorum* (Lib.) de *Bary* is a serious threat to the production of *B. napus* [38]. *S. sclerotiorum* causes the rotting of leaves, stems, and pods of *B. napus* and results in a considerable loss of seed yield around the world [83]. The main factors that promote *S. sclerotiorum* pathogenicity and colonization are oxalic acid and cell-wall-degrading enzymes secreted by the fungus. Oxalic acid not only acts as a pathogenic factor but also detoxifies *S. sclerotiorum* in the later stages of infection [84]. Our RNA-seq data and RT-qPCR results showed consistency of up-regulated *BnaSNARE*s (including *BnaKNOLLEs*, *BnaSYP121s,* and *BnaSYP122s*) in response to *S. sclerotiorum* and OA treatment.

In recent years, many researchers began to explore the signal pathways involved in the interaction between *S. sclerotiorum* and *B. napus* or other plants. They found that the disease resistance signals induced by *S. sclerotiorum* are mainly mediated by JA and ABA [85], which are also affected by ethylene and SA signaling pathways [45]. Our data showed that most of the BnaSYP1-type genes are differently induced by SA, JA, and ABA, suggesting a potential role in the interactions between *B. napus* and *S. sclerotiorum*.

Previous studies have shown that SNARE proteins often perform biological functions in the form of complexes in cells [47]. For example, PEN1/SYP121 can form tetramers with one scaffold protein SNAP33 and two vesicle-related membrane proteins VAMP721 and VAMP722, which jointly regulate the fusion process of vesicles and plasma membrane, and ultimately mediate plant disease resistance to pathogens [74]. Because SYP122 and PEN1/SYP121 are homologous proteins, the Yoichiro Fukao research group has identified through interaction genomics that SYP122 interacts with PEN1/SYP121 [27,39]. We infer that SYP122 may also interact with the above four proteins (SYP121, SNAP33, VAMP721, and VAMP722). For this reason, we analyzed the expression levels of *BnaSYP121s*, *BnaSYP122s*, *BnaSNAPs*, *BnaVAMP721*, and *BnaVAMP722/3s* during *S. sclerotiorum* infection and found that the expression levels of the several genes were highly correlated. The interaction network analysis of these proteins further proved the association between *BnaSYP121/122s* and *BnaSNAPs*, *BnaVAMP721*, and *BnaVAMP722/3s*, so we speculate that BnaSYP121/122 may form a complex with BnaSNAPs and BnaVAMP722/3s to promote the resistance of plants against *S. sclerotiorum*. However, the functional mechanism of these SNARE proteins needs to be confirmed by further experiments.

## 5. Conclusions

A total of 237 *BnaSNAREs* were identified, which is the highest number of the protein family unveiled in all species previously studied. The expansion of the *BnaSNARE* family is primarily due to polyploidization and hybridization events. Besides, RNA-seq data showed the expression of *BnaSNAREs* in leaves and roots of *B.* *napus*. The expression profiles under the influence of *S. sclerotiorum* implied the potential role of *BnaSNARE*s in mediating the resistance of *B.* *napus* against *S. sclerotiorum*. Differential expression trends were also observed following RT-qPCR under SA, MeJA, ABA, and OA treatments, signifying the important role of *BnaSNARE*s in multiple signaling pathways. Moreover, the interaction protein of *BnaSNARE* contains some defense response-related proteins, which increases the evidence that *BnaSNARE* protein is involved in plant immunity. Additionally, members of BnaSYP121/2s, *BnaSNAPs*, and BnaVAMP722/3 showed probable interaction and correlated expression profiles upon infection with *S. sclerotiorum*. Although elucidating the exact functional mechanism of these *BnaSNAREs* in biotic and abiotic conditions requires further analysis, our findings provide the first gene-family-wide survey on the expression patterns of specific *B. napus* SNAREs in pathological conditions, and these highly up- and down-regulated genes can serve as candidate genes for future investigations.

## Figures and Tables

**Table 1:** Homologous gene pairs of *SNAREs* in *B. napus*.

Homoeologous Group (A:C)	All Genes in *B. napus* ^1^	*BnaSNAREs* (All) ^2^			*BnaSNAREs* (Omit Not Categorized) ^4^		
		Number of Gene Pairs	Number of Genes	% of Genes	Number of Gene Pairs	Number of Genes	% of Genes
1:1	80.8	91	182	76.8%	91	182	89.7%
0:1	6.75	7	7	3.0%	7	7	3.4%
1:0	12.4	11	11	4.6%	11	11	5.4%
Other ratios ^3^	-	3	3	1.3%	3	3	1.5%
Not categorized	-	-	34		-	-	
Total			237	100.0%		203	100.0%

^1^ [32]. ^2^ See Table S4. ^3^ Other ratios: one of the homologous not belong to *SNAREs*. ^4^ Not categorized: genes located on random chromosomes.

## Data Availability

All data generated or analyzed during this study are included in this article and its Supplementary Information Files. The RNA-Seq data libraries were generated in this research. The sequences of *B.* *napus* and expression profiles of BnaSNAREs downloaded from the *B**. napus* genome resources are accessible via the following link https://www.genoscope.cns.fr/brassicanapus/, accessed on 26 October 2020.

## Supplementary Materials

The following supporting information can be downloaded at: https://www.mdpi.com/article/10.3390/plants11050711/s1, Figure S1: Maximum-likelihood phylogeny of SNARE proteins from *B. napus* and *A. thaliana* Branches not transformed; Figure S2: Expression profiles of *BnaSNAREs* in roots and leaves of *B. napus*; Figure S3: Lesions in the leaves of *B. napus* varieties Zhongshuang9 and 84039 at different times after inoculation with *S. sclerotiorum*; Table S1: The characteristics of SNARE genes from *B. napus*; Table S2: Conserved domains of SNAREs in *Arabidopsis thaliana* predicted by CDD-Batch; Table S3: Subcellular localizations of BnaSNARE proteins; Table S4: Homologous gene pairs of SNAREs among An and Cn sub-genomes of *B. napus* and their putative orthologs in *A. thaliana*; Table S5: Grouped number of SNARE proteins in *Triticum aestivum*, *Oryza sativa*, *Solanum lycopersicum, Arabidopsis thaliana*, and *Brassica napus*; Table S6: Twenty conserved motifs of BnaSNAREs defined by the MEME suite; Table S7: Tandem duplication events of *BnaSNARE* genes; Table S8: The estimated Ka/Ks ratio for each pair of paralog genes from *B.* *napus*; Table S9: RNA-Seq data of *BnaSNAREs* in root and leaf. Read abundance is shown in terms of reads per kilobase million; Table S10: RNA-seq expression of BnaSNAREs in leaves at 12 h and 22 h post-inoculation with *S. sclerotiorum*; Table S11: Expression profiles of *Qa-SNAREs* in resistant (Zhongshuang9) and susceptible (84039) varieties of *B. napus* under treatments with ABA, MeJA, SA, and OA; Table S12: Putative protein–protein interaction networks among BnaSYP121s, BnaSYP122s, BnaSNAPs, BnaVAMP721s, and BnaVAMP722/3s; Table S13: List of AtSNAREs interacting proteins predicted by STRING-DB; Table S14: Orthologs of AtSNAREs interacting proteins in *Brassica napus*; Table S15: Primers used in this study.
