# Supplementary material for: Genome-Wide Identification and Expression Analysis of SNARE Genes in Brassica napus
Source: Plants (Basel). 2022 Mar 7;11(5):711. doi: 10.3390/plants11050711 (PMC8912762; doi:10.3390/plants11050711)
Supplement: Supplementary file 1 [file plants-11-00711-s001.zip › Figures S1¿CS3.pdf]

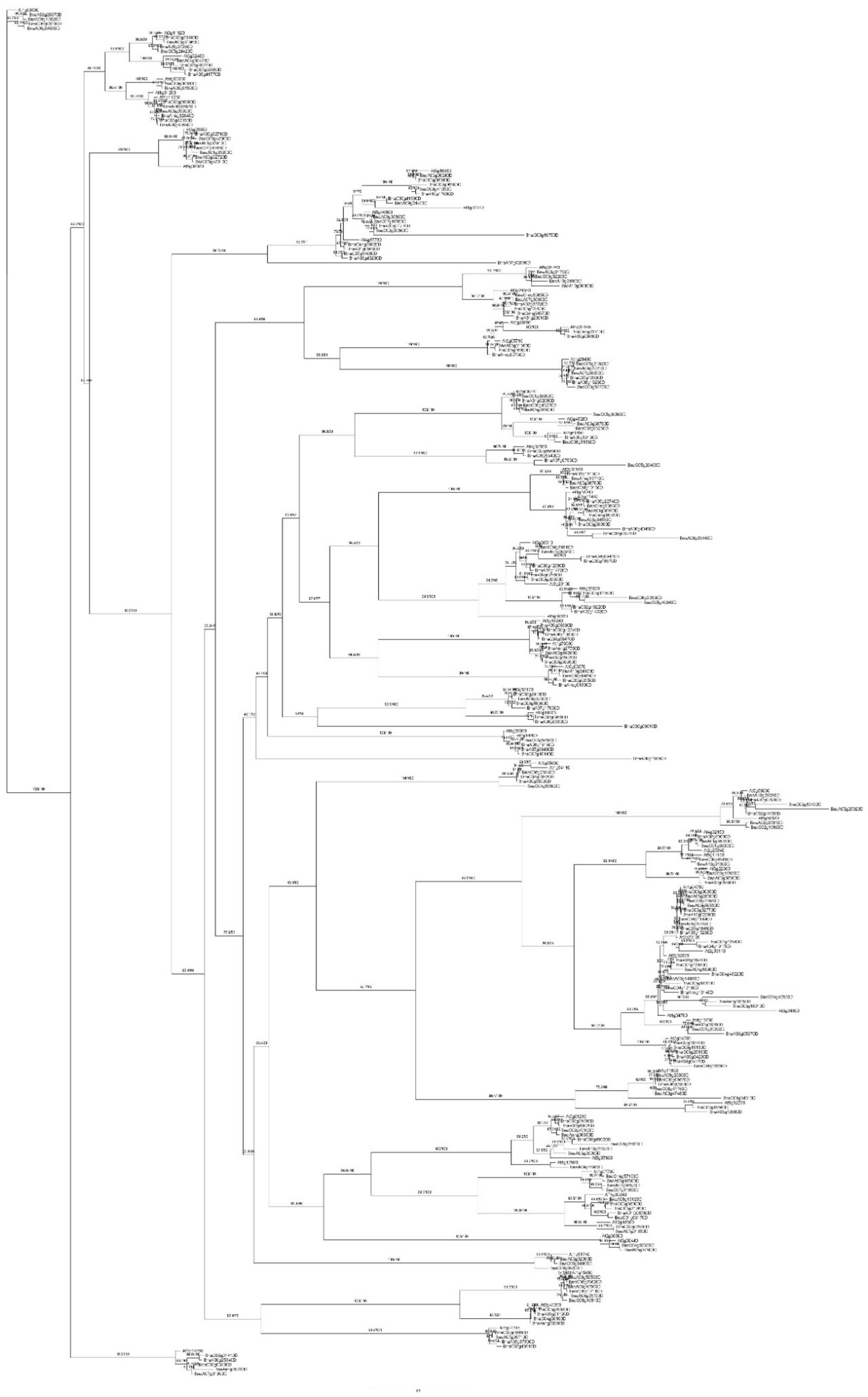

Figure S1. Maximum likelihood phylogeny of SNARE proteins from *B. napus* and *A. thaliana* Branches not transformed. SH-

aLRT and Ultrafast bootstrap values are indicated on the branches.

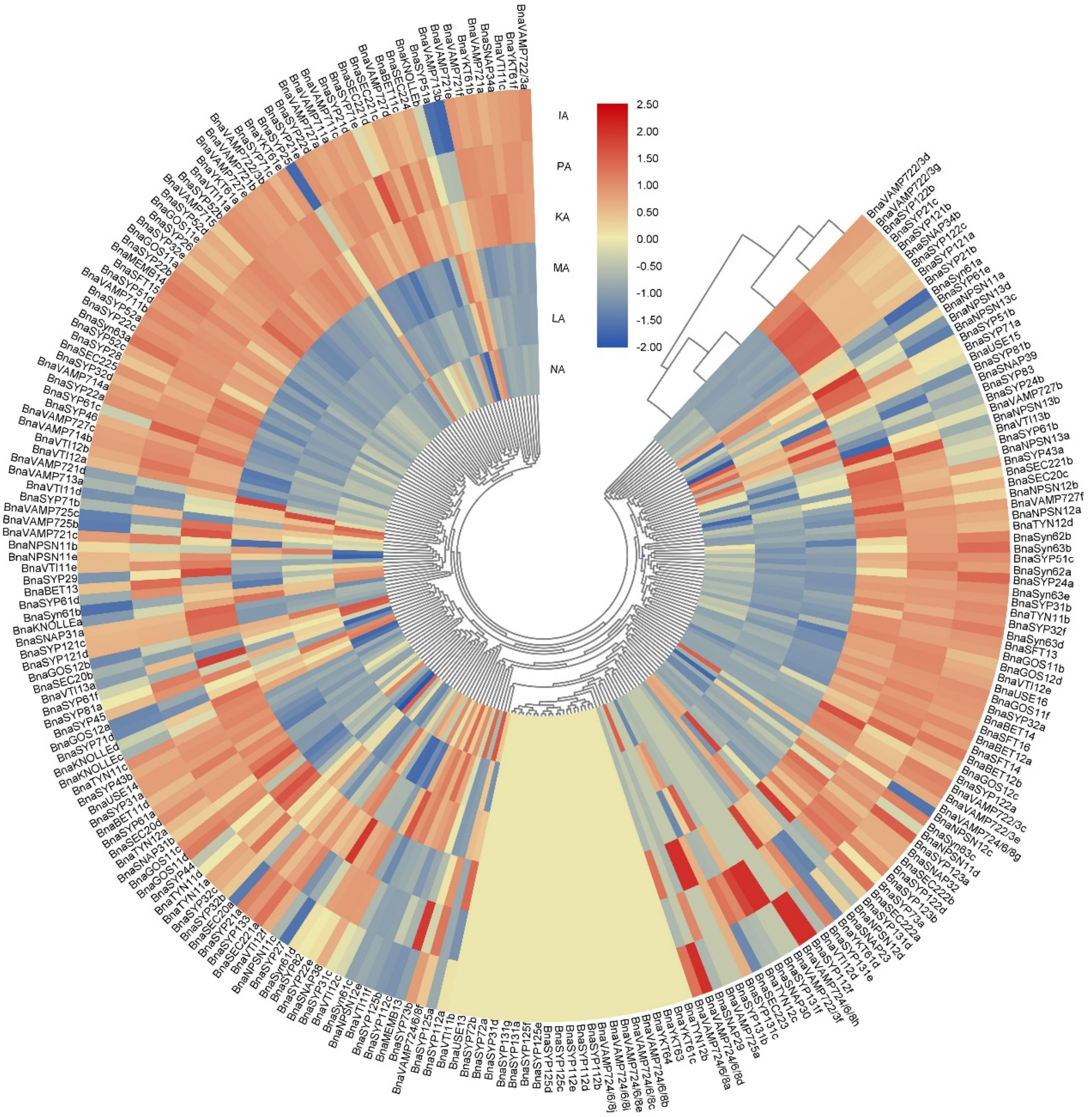

Figure S2. Expression profiles of *BnaSNAREs* in roots and leaves of *B. napus* PA, IA, KA represent *B. napus* roots replicate 1, 2 and 3 transcriptomes; LA, MA, NA represented *B. napus* leaves replicate 1, 2 and 3 transcriptomes. Data were normalized by Log2 (RPKM: Reads Per kb Per Million reads). The cluster tree of the *BnaSNARE*s based on the expression level is shown at the center and top.

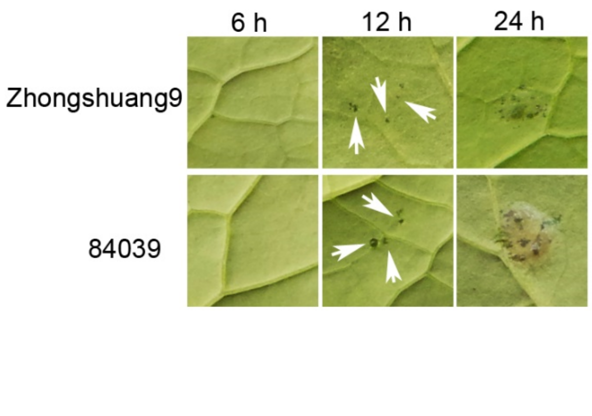

Figure S3. Lesions in the leaves of *B. napus* varieties Zhongshuang9 and 84039 at different time after inoculation with *S. sclerotiorum.*
